# Association of Physicochemical Characteristics, Aggregate Indices, Major Ions, and Trace Elements in Developing Groundwater Quality Index (GWQI) in Agricultural Area

**DOI:** 10.3390/ijerph18094562

**Published:** 2021-04-25

**Authors:** Hazimah Haspi Harun, Mohamad Roslan Mohamad Kasim, Siti Nurhidayu, Zulfa Hanan Ash’aari, Faradiella Mohd Kusin, Muhammad Khalis Abdul Karim

**Affiliations:** 1 Department of Forestry Science and Biodiversity, Faculty of Forestry and Environment, Universiti Putra Malaysia, UPM Serdang 43400, Selangor, Malaysia; mohdroslan@upm.edu.my (M.R.M.K.); sitinurhidayu@upm.edu.my (S.N.); 2 Department of Environment, Faculty of Forestry and Environment, Universiti Putra Malaysia, UPM Serdang 43400, Selangor, Malaysia; zulfa@upm.edu.my (Z.H.A.); faradiella@upm.edu.my (F.M.K.); 3 Department of Physics, Faculty of Science, Universiti Putra Malaysia, UPM Serdang 43400, Selangor, Malaysia

**Keywords:** GWQI, PCA, agricultural, Kuala Langat, Kota Bharu

## Abstract

The aim of this study was to propose a groundwater quality index (GWQI) that presents water quality data as a single number and represents the water quality level. The development of the GWQI in agricultural areas is vital as the groundwater considered as an alternative water source for domestic purposes. The insufficiency of the groundwater quality standard in Malaysia revealed the importance of the GWQI development in determining the quality of groundwater. Groundwater samples were collected from thirteen groundwater wells in the Northern Kuala Langat and the Southern Kuala Langat regions from February 2018 to January 2019. Thirty-four parameters that embodied physicochemical characteristics, aggregate indicator, major ions, and trace elements were considered in the development of the GWQI. Multivariate analysis has been used to finalize the important parameters by using principal component analysis (PCA). Notably, seven parameters—electrical conductivity, chemical oxygen demand (COD), magnesium, calcium, potassium, sodium, and chloride were chosen to evaluate the quality of groundwater. The GWQI was then verified by comparing the groundwater quality in Kota Bharu, Kelantan. A sensitivity analysis was performed on this index to verify its reliability. The sensitivity GWQI has been analyzed and showed high sensitivity to any changes of the pollutant parameters. The development of GWQI should be beneficial to the public, practitioners, and industries. From another angle, this index can help to detect any form of pollution which ultimately could be minimized by controlling the sources of pollutants.

## 1. Introduction

Groundwater is an important water resource for domestic uses, drinking, agricultural irrigation, and industrialization, whereby 2.5 billion people globally depend on the groundwater sources [1,2]. In recent years, the issue of deterioration of groundwater quality has attracted worldwide attention due to the growth of agricultural areas, massive industrialization, and urbanization. The human population is predicted to have doubled from 3.9 billion to 7.0 billion over the last decades, but the need for water resources will be increased threefold. The swift population growth, industrialization, urbanization, and the expansion of the agricultural sector all inflict pressure on the existing water resources [3]. The need for clean water sources for domestic and drinking purposes has doubled compared to the population growth worldwide [4]. Approximately 1.8 billion people worldwide are anticipated to face water shortages and it is predicted two-thirds of the population will experience water stress by 2025 [5]. 

Moreover, the depletion of surface water due to seasonal changes such as climate change and prolonged drought seasons affect the storage of surface water. Malaysia faced a prolonged dry season due to El Nino in 2019 and several states were affected, especially in Peninsular Malaysia. The utilization of groundwater is significantly important due to the water shortage in dry periods, the water demand due to population expansion and the deterioration of the surface water quality. The negative impact due to water shortages contributes to the main problems to the development of society, the urban development, and the basic life of the people [6,7]. Therefore, water shortage issues require alternative water resources to preserve water sustainability and for development of socioeconomic. 

The quality of surface water is a sensitive issue where its emphasis on environmental sustainability, social welfare, and long-term economic development. The awareness of water pollution rises attention worldwide in recent years. Therefore, the sustainability of water quality needs to be implemented to achieve good management of water resources. Furthermore, a suitable index is required to assess the quality of water. The water quality index is one form of method or indicator in shortening complex water quality data and making it easier in communicating with general society. To date, Malaysia currently uses the existing Water Quality Index (WQI) (by the Department of Environment, DOE) and Drinking Water Quality Standard (by the Ministry of Health, MOH). 

As an alternative of the clean water source, groundwater also needs the creation of an index to assess the level of quality. According to [8], the lack of creation of a groundwater quality standard in Malaysia and the development of standardization for contamination of groundwater and land have become gaps in the study of the present status of groundwater in Malaysia. The assessment of hydrogeochemical properties of groundwater quality is crucial to sustaining the use of fresh groundwater aquifers for domestic, agricultural, and industrial utilization. Hence, the GWQI is a significant element in water resource management. By developing the index, the complex expressions of groundwater variables can be simplified [9]. Furthermore, the GWQI is defined as a dimensionless number that combines the multiple variables of groundwater quality into a single number of standard values to the rating curves and simplifies the interpretation of the data monitoring [10]. 

In the context of environmental indices, the first water quality index was proposed by Horton in 1965 [11]. In 1970, Brown developed a water quality index which was later improved by Deininger [12]. A novel kind of environmental index was introduced by Steinhart in 1982 to gather the trends and the status of technical information in the US/Canada ecosystem of the Great Lakes [13]. Later, several types of water quality indexes were developed covering the aspects of drinking, river, marine, irrigation and recreation. Consequently, an index for determining the quality of groundwater is also needed in the environmental assessment to ensure its good quality. 

Multivariate statistical techniques were used to determine the dominant parameters in the groundwater quality index. Principal component analysis (PCA) is the most prominently utilized method in hydrochemistry studies by normalizing the variables in the dataset and employing a correlation matrix [14]. PCA is preferable in scientific studies because this analysis can reduce the dimensionality of a dataset while maintaining the characteristics of variables which contribute to the respective variation [15,16,17]. 

The development of the GWQI needs to consider suitable parameters. The groundwater mainly consists of a series of major ions [18]. According to the previous studies, the suitable parameters to determine the quality of groundwater consist of physical and chemical parameters. The physicochemical parameters include temperature, electrical conductivity, pH, salinity, turbidity, total dissolved solids (TDS) and dissolved oxygen (DO) [19,20]. Furthermore, seven important parameters which are responsible for 95% of groundwater analysis comprise major ions including magnesium (Mg), calcium (Ca), sodium (Na), potassium (K), sulfate (SO_4_^2-^), chloride (Cl^−^) and bicarbonate (HCO_3_^−^) [21,22,23]. 

Hence, these parameters are considered as the basic qualities of groundwater and approximately more than 40% of the groundwater studies in Malaysia are associated with these parameters [19,24,25,26,27]. Meanwhile, this study considered the parameters from the four important groups of parameters which were physicochemical characteristics, aggregate indicator, major ions, and trace elements. The suitable parameters to be assessed in the GWQI were selected from these groups of parameters. The potential of groundwater sources in agricultural areas needs to be assessed to ensure its suitability and safety for agricultural use. Hence, the aim of this study is to develop a suitable GWQI for domestic purposes, specifically in a tropical climate country such as Malaysia. The study areas focused mainly on the agricultural areas that uses groundwater wells as their alternative water sources.

## 2. Materials and Methods

### 2.1. Study Sites

This study was conducted in Kuala Langat, a large district in Selangor, Malaysia. Thirteen groundwater wells were chosen and categorized into two sub-areas, Northern Kuala Langat, and Southern Kuala Langat. The study area represented a part of the Langat River Basin. The groundwater wells in Northern Kuala Langat were labelled as BKLTW12, MW01, MWD4, BKLEW2, MW05, BKLTW19, MWD2 and MWD5 and are physically shallow, and approximately 4 to 35 m depth. Meanwhile, the groundwater wells, BKLTW16, J7-1-4, BKLTW11, BKLTW15 and BKLEH29 located in the Southern Kuala Langat was deeper (more than 60 m depth). The wells then were categorized into three different well depths which were shallow, intermediate, and deep [28].

**Figure 1:**
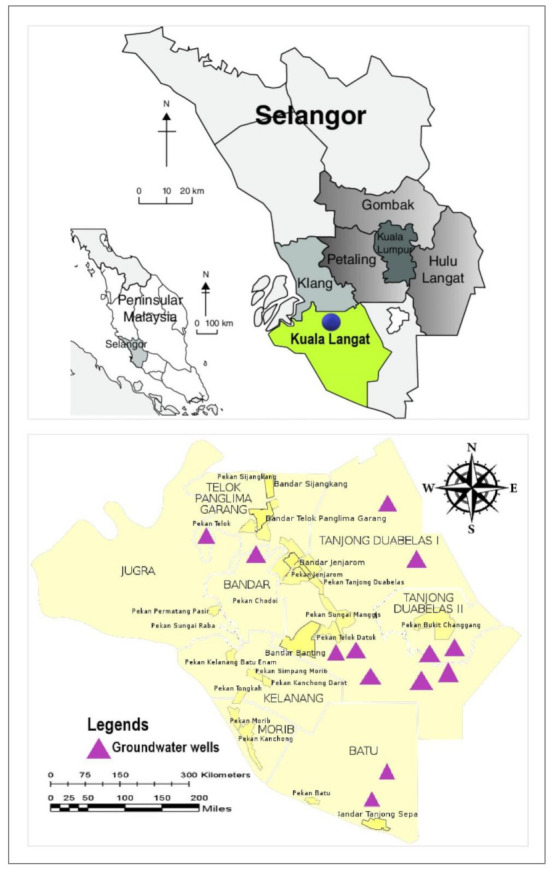
The sampling stations of groundwater wells in Kuala Langat.

The significance of the study area represented the objective of the study which focused on the groundwater sources in agricultural areas. Kuala Langat is known as an agricultural hub and prominent agritourism industry center. The study area located in the oil palm plantation area which is the major crop in Kuala Langat. Kuala Langat also has tea plantations, mixed farming, and many vegetable cultivations projects. The geology of the area is represented by a quaternary geology consisting of marine and continental deposits such as silt, sand, and peat with minor gravels. The groundwater wells in this study were in alluvial areas. The area of the groundwater recharge was from the hilly areas and the mountains upstream. Generally, the aquifers were extensively disseminated in the flat lowlands. Figure 1 shows the locations of the thirteen tested groundwater wells in Kuala Langat.

### 2.2. Groundwater Sampling

The sampling was conducted from February 2018 to January 2019. The phases of sampling collection were carried out quarterly within the sampling period. Sampling sessions were conducted during the dry and wet seasons, respectively. The groundwater sampling procedure and analysis were carried out following the guidelines established by APHA [29]. The physicochemical characteristics, aggregate indicators, major ions, and trace elements of groundwater were evaluated. The other in-situ parameters analyzed includes temperature and the total dissolved solids using a calibrated 6P Ultrameter (Myron L Company, Carlsbad, CA, USA). An Orion 3-star Portable pH meter (Thermo Scientific, Waltham, MA, USA) was used to measure the pH, while the YSI 30 Salinity and Conductivity meter (Yellow Springs, OH, USA) was used to measure the salinity and electrical conductivity. Turbidity was measured using a Thermo Orion AQ4500 Turbidity meter (Waltham, MA, USA). Measurement of alkalinity using a titration method followed APHA [29] guidelines. Meanwhile, dissolved oxygen was measured in the laboratory using a YSI 30 Dissolved Oxygen meter (Carlsbad, CA, USA). The groundwater samples were pumped using submersible groundwater pumps and a groundwater level meter was used to measure the groundwater depths before and after the sampling process. During the sampling procedure a purging pump was used to remove the stagnant groundwater for approximately 15 to 30 min to ensure the groundwater samples did not contain unnecessary elements [28,30].

### 2.3. Parameter Selection

The selection of dominant parameters in the GWQI was based on the literature review from previous studies across the world, including Malaysia, and the recommendations from the Mineral of Geoscience Department Malaysia (JMG) which is the authoritative agency for groundwater studies in Malaysia [18,31,32,33,34]. Thirty-four parameters were considered in the development of GWQI, which were differentiated into four subgroups: physicochemical characteristics, aggregate indicators, major ions, and trace elements. Table 1 shows the thirty-four parameters considered in the development of GWQI according to the different important groups.

The dominant parameter for the GWQI was identified using the principal component analysis (PCA) method. The number of samples used in the statistical analysis (135) of the data set followed the requirements of PCA. For conducting PCA it is recommended that the sample size exceed 100 and a sample size of less than 100 is considered unsuitable [35]. In PCA, the selection of dominant parameters is divided into three phases which are the preliminary analysis for the selection of dominant parameters, selection of dominant parameters according to subgroups, and finally, the selection of dominant parameters. The preliminary analysis was considered as the first stage in identifying important parameters to develop the groundwater quality index. The preliminary analysis is essential in terms of observing the relevant connections between parameters. The analysis for the second stage of PCA focused on the distinguished subgroups of parameters. The subgroup included physical characteristics, aggregate indicators, major ions, and trace elements. The dominant parameters of the second stage analysis proceeded to the final PCA analysis.

The development of the GWQI implemented a proportional technique to determine the subindex and the interquartile technique to identify the range of index scores for each parameter. The PCA was carried out using the SPSS 25 statistical software designed by International Business Machines (IBM), New York, USA for the important parameter selection. The GWQI formulation was carried out using Microsoft Office Excel 2016 (Microsoft Corporation, Washington, DC, USA).

### 2.4. Normality Testing

The important criterion to be considered before the identification of the important parameters in the GWQI was normality testing. Normality testing was conducted for all thirty-four parameters to obtain the normal data tabulation for conducting the PCA. PCA is a multivariate analysis method that is the most prominently utilized by researchers and scientists in Malaysia and other countries to evaluate the hydrochemistry of their respective study areas [14]. PCA identifies the most significant parameters in the data set and reduces the data complexity to allow a better understanding of the variation among the parameters [36]. 

**Figure 2:**
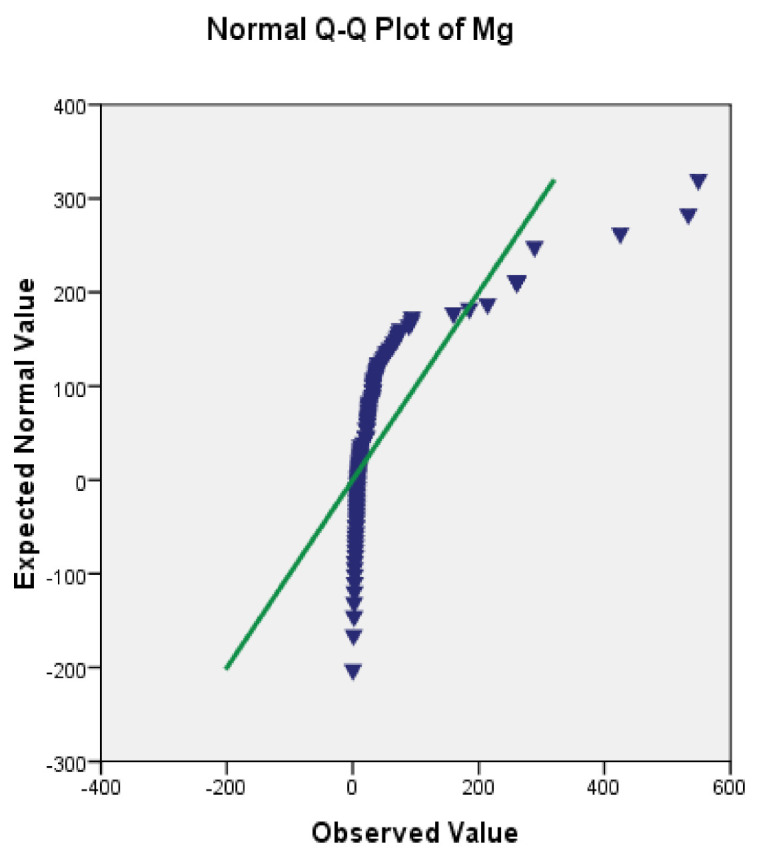
Q-Q plot before log transformation for magnesium.

The testing of normality was performed for the thirty-four parameters before the PCA analysis could be carried out. The results obtained from the PCA would be accurately calculated if the data were normally distributed. A Q-Q plot analysis was performed for the normality testing in this study. The Q-Q plot analysis showed clear views of the normality of the data distribution and illustrated it in the graphic features. The Q-Q plot analysis showed for example that the magnesium concentration in groundwater was not normally distributed based on the curve pattern which was not parallel with a straight line as shown in Figure 2. 

From the Q-Q plot analysis, it was found the data transformation was appropriate to ensure that the data was in a normal distribution [36]. The standard deviation for the original data showed high values compared to the data that has been transformed. The value of the standard deviation can significantly affect normal data distributions. A small value of the standard deviation can significantly obtain a high probability value or *p*-value [37]. Therefore, to ensure the data is normally distributed, the standard deviation is supposed to have a small value. A comparison of the standard deviation between the original data and the transformed data is shown in Table 2.

**Figure 3:**
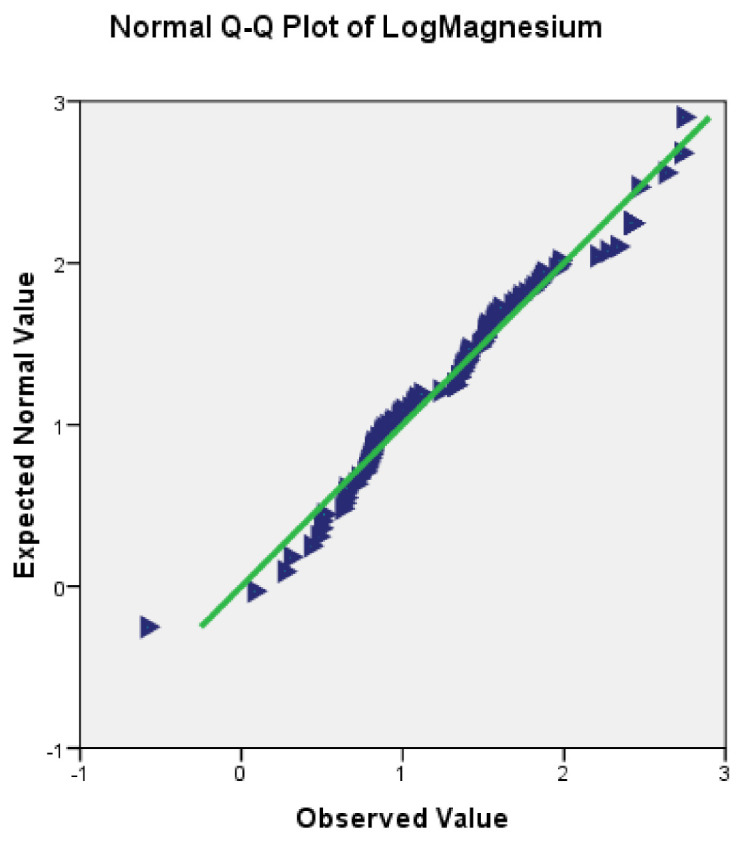
Q-Q plot after log transformation for magnesium.

Therefore, the reanalysis of the Q-Q plot after this transformation showed that the data were distributed in a straight line. This means that this transformation succeeded in transforming the data to be normally distributed. Figure 3 shows the example of the Q-Q plot analysis for magnesium which now shows the normal distribution after data transformation.

Meanwhile, the normality of the data can be proven using Kolmogorov-Smirnov testing. A *p*-value less than 0.05 shows the data is not normally distributed. By transforming the data using log-transformation, the Kolmogorov-Smirnov test reanalysis produced a *p*-value larger than 0.05 which means that the data is distributed normally. Table 3 shows the normality testing using Kolmogorov-Smirnov and shows the magnesium was less than 0.05 and the data not distributed normally. After the log transformation, Table 4 shows the data were distributed normally where the *p*-value was 0.666 which is more than 0.05.

### 2.5. Development of the Groundwater Quality Index

The GWQI in agriculture can be developed after the important parameters have been finalized accordingly from the four groups of parameter classes, namely physicochemical parameters, aggregate indicator parameters, major ions, and trace elements. The development of the GWQI occurs in the following stages:

#### 2.5.1. Minimum and Maximum Value

The concentration of the groundwater in the undisturbed forest was chosen as the minimum limit for the dominant parameters in the GWQI. The minimum range in GWQI was obtained from a pristine place such as forest which less vulnerable to the pollution. The slower recharge rates of groundwater in the forests significantly contributed to the delay in the deterioration of groundwater quality. Furthermore, the Malaysian Ministry of Health had fixed maximum limits for certain metallic ions. Therefore, this value was used for the maximum limit in this study. For the other six index indicators, the maximum permissible limit from the established guidelines such as the World Health Organization’s Drinking-Water Quality Standard [38], Water Quality Standards of Environmental Protection Agency [39] and Indian Standard for Drinking Water [40] and United Nations Environment Program were [41] used as the maximum limits in this study.

#### 2.5.2. Sub Index Values based on Proportional Analysis

The GWQI subindex was determined using proportional analysis to determine the composite values. The development of GWQI in this study emphasized a statistical approach was different from other GWQIs. The most common methods use standardization techniques by creating an empirical rating curve from a surveys and interviews approach to obtain the sub-index values [11,32,33,34]. The index developed by Horton [11] used aggregations such as arithmetic, harmonic, geometric, and weighting factors to identify the subindex. This weighting and aggregation technique was also used by Stigter [34] and Saeedi [33] for their groundwater quality index development. In this study, the subindex determination using the proportional analysis only can be calculated after the minimum and the maximum ranges have been identified in the previous step. The range in the proportional analysis will be from 0 to 100 for each parameter, where 100 represents excellent quality. Therefore, for any concentration of the parameters which exceeded the maximum limit which has been set in this groundwater quality index, the value is defined as zero. The calculation of the subindex value based proportional analysis can be defined as:

(1) $$\;\mathrm{Sub}\;\mathrm{index}=\frac{\mathrm{Maximum}\;\mathrm{value}-\mathrm{Average}\;\mathrm{parameters}\;\mathrm{concentation}}{\mathrm{Maximum}\;\mathrm{value}-\mathrm{Minimum}\;\mathrm{value}}\times 100$$

#### 2.5.3. Range of Index Scores Based on Interquartile Analysis

The interquartile range (IQR) was calculated based on the difference between the upper and lower quartiles of Q3 and Q1. The measurement of the interquartile range (IQR) is a variability measurement where the data is divided into quartiles. The purpose of finding the interquartile in the groundwater quality index is to set up the classes for classifying the level of groundwater quality. For example, the index range is from 0–100, so if the index value is 100, then it can be confirmed that the quality is excellent and if the value is 0, then it can be confirmed that the quality is poor, but the interpretation is quite hard if the value is something like 35 or 65, for example. The Q3 and Q1 values were determined from the frequencies of the dataset for the concentration of dominant parameters in groundwater samples using the descriptive analysis which obtains from the SPSS analysis. Therefore, the IQR was calculated using Equation (2):

(2) Q3−Q1=Interquartile Range

The classification of the groundwater quality can be determined through the inter-quartile analysis. The maximum values for all dominant parameters was based on the maximum recommended levels in raw water stipulated by the Ministry of Health and the World Health Organization. Basically, the quartile value was determined to represent a class of the water quality as Class I, II, III, and IV. The quartile value can be obtained using the following equation:

(3) $$\mathrm{New}\;\mathrm{Quartile}\;=\frac{\mathrm{Maximum}\;\mathrm{value}-\mathrm{Different}\;\mathrm{Quartile}}{\mathrm{Maximum}\;\mathrm{value}}\times 100$$

## 3. Results and Discussion

### 3.1. Principal Component Analysis

In PCA, the selection of dominant parameters in the preliminary analysis is considered as the first stage in identifying important parameters to develop the groundwater quality index. The preliminary analysis was essential in terms of observing the relevant relations between parameters. The main purpose of conducting the preliminary analysis was to determine the data structure of each of the 34 parameters besides determining the initial correlation among variables which could indicate whether the dimensionality of the variables could be reduced. 

Table 5 summarizes the total variance and rotated components matrix for this preliminary analysis. It was observed that seven components had eigenvalues greater than 1. Therefore, a seven components solution explained 75.121% of the cumulative total variance. A varimax orthogonal rotation was employed to aid interpretability and to further identify the factors responsible for each one. In this case, the dataset relating to a preliminary analysis showed a strong loading for chloride, electrical conductivity, sodium, potassium, magnesium, total solids, and salinity represented by PC1 which explained 20.04% of the total variance. 

The analysis for the second stage of PCA focused on the distinguished subgroups of parameters. The subgroups included physical characteristic, aggregate indicator, major ions, and trace elements. The purpose of this analysis process was to observe the relevant aspects of the subgroups. Meanwhile, in the final stage of PCA, the dominant parameters can be finalized from the result of PCA for all the subgroups analysis. Therefore, from the PCA results, the eigenvalues greater than 1 determined as dominant parameters. There were 16 parameters from the distinguished subgroups of physicochemical characteristics, aggregate indicator and major ions that were considered in the final PCA.

The PCA results for the final stage of dominant parameter selection revealed seven components that had eigenvalues greater than 1 which explained 43.33, 21.66, 8.54, 6.34, 4.26, 3.37 and 3.05% of the total variance, respectively. Therefore, the four components solution explained 90.65% of the cumulative total variance. A varimax orthogonal rotation was employed to aid interpretability and to further identify the factors responsible for each one. In this case, the data set relating to the final PCA showed strong loadings for electrical conductivity, chemical oxygen demand, magnesium, calcium, potassium, sodium, and chloride represented by PC1 which explained 36.24% of the total variance. The final PCA revealed the dominant parameters in GWQI consisted of major ions that occurred from the rock weathering process and depended on the geographical characteristics [42,43]. Electrical conductivity also appeared in GWQI and referred to the occurrence of high alkalinity and electrical conductivity in the groundwater due to the high concentrations of most cations [44,45,46,47]. Meanwhile, the absence of trace elements in the selection of dominant parameters for GWQI caused to the chemical elements in groundwater such as nitrate, nitrite, phosphate, and ammonia are less abundant in groundwater as they are easily degraded by microorganisms at a high degradation rate [48,49]. 

Moreover, according to the Department of Agriculture Malaysia [50], the application of fertilizer and manure in the study area has generally followed the Malaysian Farm Certification Scheme for Good Agricultural Practice (SALM) certification regulations. Therefore, due to this implementation, the chemical parameters derived from agricultural practices do not accumulate significantly on land and in water bodies, and pollution was mitigated. Table 6 shows the values of total variance explained by the final analysis and rotated components matrix for preliminary analysis. Table 7 shows the parameters considered as the dominant parameters in the groundwater quality index. Table 8 shows the minimum and the maximum limits for each dominant parameter.

Table 9 shows the calculation and the proportional analysis for each parameter selected in the groundwater quality index and the proposed subindex value. The calculation of proportional analysis was referred to the formula of equation 1 for the purposes to determine the value of the subindex. 

### 3.2. Graphical Features of the Index

**Figure 4:**
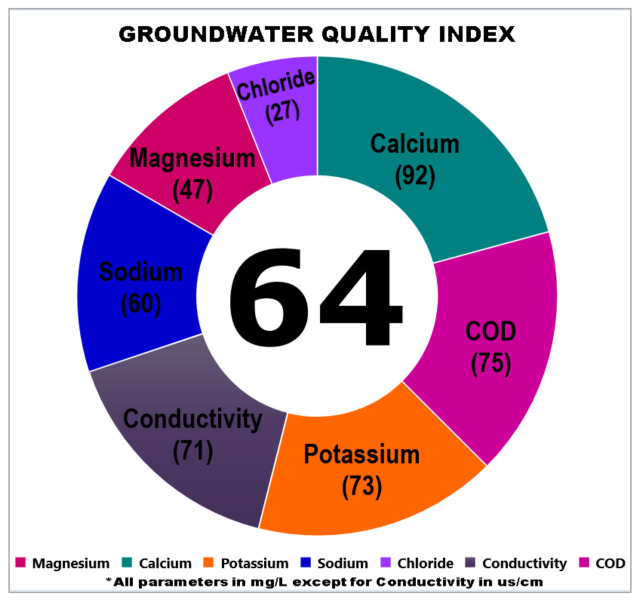
GWQI in graphical features.

The subindex determination for all dominant parameters in the previous step was computed into one figure by calculating the average of all the parameters to determine a single unit for the index. The GWQI in the current study showed the index was 64 which represents the quality of groundwater in the Kuala Langat agricultural areas. The borders of each section in the graphical index began with the parameters with the highest subindex value and followed by the lower values. The size of the graph border was also affected depending on the value of the subindex for each parameter where the broad section representing the high subindex value and followed by the small section for the low subindex value. However, this index was not complete enough to determine the goodness of the water as there was no classification of the water. Therefore, the next step was developing the index classification process. The graphical features of the index of the groundwater quality in Kuala Langat are shown in Figure 4. Table 10 shows the results of the IQR values for all dominant parameters. 

The range of index scores for the GWQI using an interquartile technique has been calculated in detail. The determination of quartile for the range of index scores of GWQI has been shown and magnesium has been used for an example. The calculation of quartile values for other dominants parameters also used the same technique for the purposes to obtain the range of index scores of groundwater quality.

*Step 1:*

$$Q3-Q1=\mathrm{Interquartile}\;\mathrm{Range}\;\left(\mathrm{IQR}\right)$$

48.26−6.8=41.46

*Step 2:*

$$\frac{\mathrm{Maximum}\;\mathrm{value}}{\mathrm{IQR}}=\mathrm{Actual}\;\mathrm{Quartile}\;\mathrm{Borders}\;\left(\mathrm{AQB}\right)\;$$

$$\frac{150}{41.46}=3.42$$

*Step 3:*

The new quartile borders were obtained from the actual quartile borders. The actual quartile borders need to be divided by 3 for the purpose of creating the first, second, and third quartile. Therefore, the new quartile borders need to create for three quartiles and the calculation was simplified as below.

$$\mathrm{New}\;\mathrm{Quartile}\;\mathrm{Borders}\;\left(\mathrm{NQB}\right)\;=\frac{\mathrm{Actual}\;\mathrm{Quartile}\;\mathrm{Borders}\;\left(\mathrm{AQB}\right)}{3}\;$$

$$\mathrm{NQB}=\frac{3}{3}=1$$

Therefore, the new quartile borders have represented as below.

NQB1=1

NQB2=2

NQB3=3

*Step 4:*

$$\mathrm{Quartile}\;\mathrm{Different}\;\left(\mathrm{QD}\right)=\mathrm{Interquartile}\;\mathrm{Range}\;\left(\mathrm{IQR}\right)\times \mathrm{New}\;\mathrm{Quartile}\;\mathrm{Borders}\;\left(\mathrm{NQB}\right)$$

$$\left(\mathrm{QD}1\right)=41.46\times 1=41.46$$

$$\left(\mathrm{QD}2\right)=41.46\times 2=82.92$$

$$\left(\mathrm{QD}3\right)=41.46\times 3=124.38$$

*Step 5:*

$$\mathrm{New}\;\mathrm{Quartile}=\frac{\mathrm{Maximum}\;\mathrm{value}-\mathrm{Quartile}\;\mathrm{Different}\;\left(\mathrm{QD}\right)}{\mathrm{Maximum}\;\mathrm{value}}\times 100$$

$$\mathrm{Quartile}\;1=\frac{150-41.46}{150}\times 100=72.36$$

$$\mathrm{Quartile}\;2=\frac{150-82.92}{150}\times 100=44.72$$

(4) $$\mathrm{Quartile}\;3=\frac{150-124.38}{150}\times 100=17.08$$

The determination of IQR was essential to classify the quartile into different classes. The quartile identification was also calculated for seven important parameters. The quartile calculation obtained the different results for each important parameter. Therefore, to obtain a more uniform result for the groundwater classification, the range of classification values was calculated for average value from the seven important parameters. The quartiles formed into four different classes were Class I, Class II, Class III and Class IV. The uniformity of the range of index scores for distinct classes in this study implements from the descriptions of basic statistical for data preprocessing in the justification of analytical foundation [51]. Table 11 shows the final range of index scores for the groundwater quality index. Table 12 shows the distinct classes referring to the range of index scores to determine the groundwater quality index.

### 3.3. Groundwater Classification and Description

The computed GWQI scores for domestic groundwater utilization were classified into four types which were excellent, good, fair, and poor according to the range of index scores. The groundwater classification is explained in Table 13. The groundwater sources in Kuala Langat showed an index value of 64 and are defined as Class II where the groundwater classification is good. 

Meanwhile, the use of GWQI scores to determine the suitability of groundwater utilization for domestic purposes according to the groundwater classes has also been described. A high index indicates a good groundwater quality and low pollutant concentrations. The evaluation of the index value in the current study showed the groundwater sources were considered as good quality and suitable for use as alternative water sources, especially for domestic purposes. Table 14 shows the groundwater description for each groundwater classification.

### 3.4. Verification of the Applicability of the Index

The verification process of this index was carried out by determining the groundwater quality in the agricultural area near Kampung Banggol, Kota Bharu, Kelantan which consisted of paddy fields and oil palm plantations. Groundwater samples were collected from five groundwater wells monitored by the Department of Irrigation and Drainage (JPS), Kota Bharu Kelantan. The largest groundwater abstraction area was identified in Lower Kelantan Basin and categorized as the most active agricultural area in Malaysia [52]. The method for verification of the applicability of the GWQI in this study however shows a limitation on the selection of the suitable area to evaluate the quality of groundwater. 

**Figure 5:**
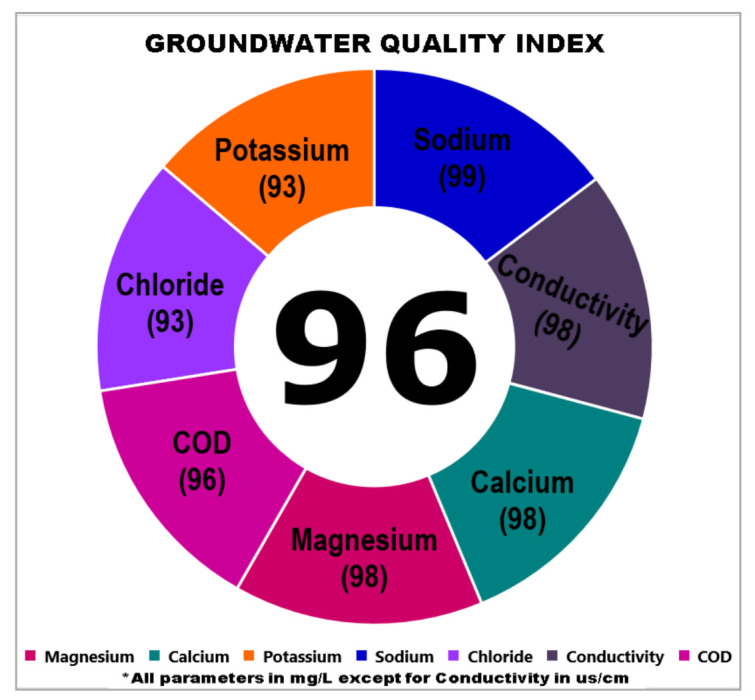
GWQI in Kelantan agricultural areas.

Nevertheless, according to the dominant parameters selected in the GWQI, there has significant reliability to evaluate the quality of groundwater due to the concentration of major ions which are generally abundant in groundwater [18,53,54,55,56]. Consequently, the developed GWQI can be applied in different areas, especially which represent the agricultural or coastal areas. Therefore, the groundwater wells in Kota Bharu represent a suitable area for verification of the applicability of groundwater quality index. It was found that the index value was 98 which means that the quality was excellent. Based on the observation, the wells in that area were suitably used by the surrounding community as an alternative clean water supply. The GWQI in the Kelantan agricultural area is presented in Figure 5. 

### 3.5. Sensitivity Analysis of the Groundwater Quality Index

A sensitivity analysis was implemented to evaluate the statistical verification of the index and measure the impact of changes on the index expression for index verification purposes. The sensitivity analysis was important in the development of the index due to the flexibility and the ease of the index computation [31]. The changes in the cumulative index value show any modification in the average parameter concentration. For example, the cumulative index value in the groundwater samples in Kuala Langat was indicated as 64. Therefore, to test the sensitivity of the index, the concentration of magnesium was modified to an extreme value and set as 200 mg/L. This value exceeded the permissible limits of magnesium in the raw water which is stipulated by the [38,39,40] as 150 mg/L. As a result, the subindex value for magnesium appeared as a negative value which was –33.56 and the subindex for magnesium was automatically set as a zero value.

**Figure 6:**
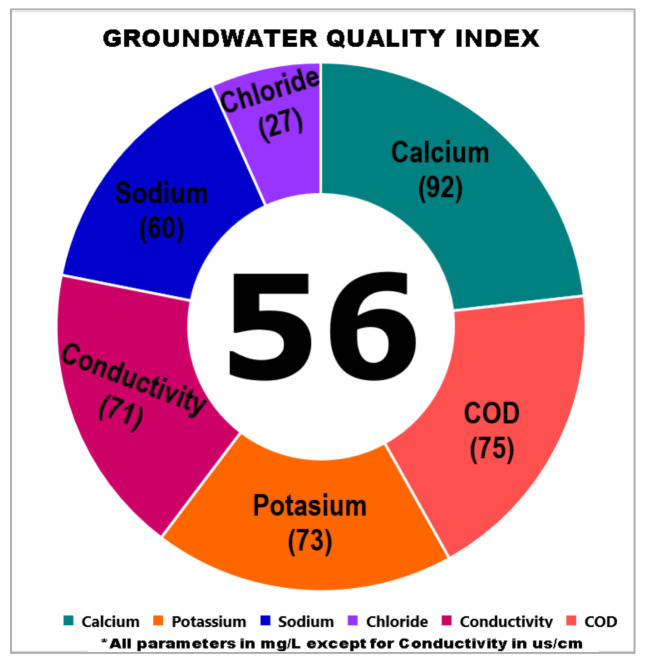
Change in the index value as the Mg concentration increases.

The magnesium concentration change from 79.5 mg/L to 200 mg/L therefore affected the cumulative index value and increasing the magnesium concentration changed the value of the GWQI to 56. Due to the zero subindex value, the concentration of magnesium was not included in the graphical index, but the groundwater was still classified as good quality according to the range of index scores and thus suitable for domestic utilization. The GWQI therefore showed significant sensitivity to any parameter changes. The sensitivity level can be determined through the increment or the decrement in the percentage of the index value. Therefore, the GWQI is reliable enough to be used to determine the quality of groundwater. Table 15 shows the calculation of the subindex by modifying the concentration of magnesium to 200 mg/L. Figure 6 also shows the graphic index of the GWQI which decreased to 56.

**Figure 7:**
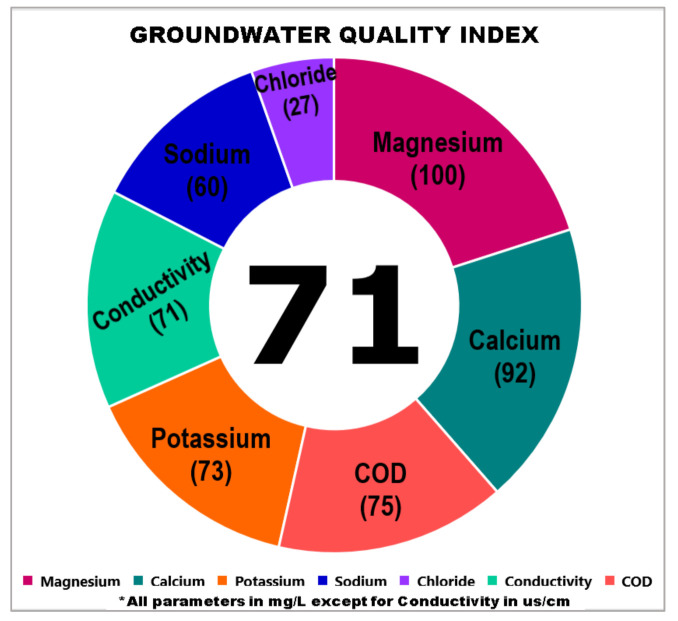
Change in the index value as Mg concentration decreases.

Meanwhile, the sensitivity of the index was also tested by decreasing the concentration of the parameter into the lowest value of 0.24 mg/L which found in the J2-1-2 groundwater well in Kuala Langat. The reduction of the magnesium concentration also affected the subindex value that changed from 47 to 100. Therefore, the cumulative index showed an increment from 64 to 71. Table 16 shows the calculation of the subindex after modifying the concentration of magnesium to 0.24 mg/L. Figure 7 also shows the graphic index of the GWQI which increased to 71.

## 4. Conclusions

The main contribution of this study is to develop a GWQI for agricultural areas to determine whether the groundwater is suitable to be an alternative clean water supply. The development of the GWQI was focused on the agricultural areas to identify the dominant parameters that could be the main indicators in the index to assess the quality of the groundwater. Referring to the results obtained, the application of fertilizers and manures from agricultural practices does not significantly show any degradation of the quality of groundwater.

Principal component analysis (PCA) was used to determine the dominant parameters in the groundwater quality index. Seven dominant parameters were selected as index indicators to assess the quality of groundwater in the agricultural area, which are magnesium, calcium, potassium, sodium, chloride, electrical conductivity, and chemical oxygen demand. Major ions were more dominant parameters that explained the groundwater source as these ions are generally abundant in groundwater sources. The abundance of major ions in the groundwater sources in Kuala Langat potentially contributes to the high electrical conductivity in the groundwater. Meanwhile, COD is an indicator of the capacity of water to consume oxygen during the decomposition and oxidation process of the major ions in the groundwater.

The GWQI used graphical features to describe the quality of the groundwater. The graphical index is user friendly and easier to comprehend. The GWQI comprises four categories named Class I, II, III, and IV in this study. Therefore, each class defines water as Excellent, Good, Fair, and Poor according to each class to determine the quality of groundwater. The GWQI has been verified for the purpose of testing whether the index is suitable to be used in different agricultural areas. Groundwater wells in Kampung Banggol, Kota Bharu, Kelantan were selected to verify the GWQI due to the fact this area is the prominent agricultural hub in Malaysia and is located near to the coastal areas. Besides, the groundwater sources have been used for many years ago in Kelantan for domestic and irrigation purposes on a big scale. The sensitivity of the GWQI index also has been analyzed and it shows high sensitivity to any changes in the pollutant parameters so the results from this index are reliable and significant.

The GWQI can be used to assess the quality of groundwater especially in locations which present similar characteristics like Kuala Langat, Selangor and Kota Bharu, Kelantan where this area located near the coastline and consists of agricultural areas. The development of GWQI is beneficial to the public, practitioners, and industries, and from another angle, this index can help to detect any form of pollution. Therefore, groundwater pollution can be controlled by detecting the source of the pollutant. 

Regarding the groundwater sources in Kuala Langat, Selangor, and Kota Bharu, Kelantan, it can be concluded that the groundwater from both areas is very suitable for clean water and domestic uses such as washing and bathing. Due to a variety of unforeseen factors such as drought, water rationing, population expansion, and surface water pollution, the groundwater sources are suitable as an alternative to accommodate the water necessity. Therefore, the groundwater sources were evaluated in the current study wherein Kuala Langat, Selangor, and Kota Bharu, Kelantan potentially can be utilized as a source of clean water for domestic uses. 

As an addition for the recommendation in the future study, the assessment of the geological analysis is necessary to determine the influences on different ion concentrations in groundwater. The soil types and characteristics play important roles to help identify the dominant elements in groundwater due to the thickness of hard rock, soil type, and the characteristics of rock and aquifer show the different accumulation of elements.

A detailed assessment regarding the land uses for agricultural areas is required to determine the substances potentially derived from agricultural practices that might permeate into groundwater. The fertilizer application from agricultural practices that affect the quality of groundwater is difficult to predicted unless a study on the soil is done. Therefore, a good agricultural practice system in agricultural areas can mitigate the contamination of groundwater sources.

Moreover, the clinical testing and health study details are also recommended for the purposes of determining if groundwater is a suitable alternative for drinking water. The use of groundwater for drinking purposes requires a detailed assessment in terms of evaluation of parameters to establish whether it is safe for human consumption as recommended by the Ministry of Health.

## Figures and Tables

**Table 1:** Thirty-four parameters considered in the development of groundwater quality index.

Aggregate Indicator	Physicochemical Characteristics	Major Ions	Trace Elements
BOD	Dissolved Oxygen	Magnesium	Aluminum
COD	Temperature	Calcium	Arsenic
	Total Solids	Potassium	Barium
	Total Dissolved Solids	Sodium	Cadmium
	Turbidity	Bicarbonate	Copper
	Electrical Conductivity	Chloride	Iron
	Salinity	Sulfate	Manganese
	pH		Strontium
	Ammonia		Silica
	Ammoniacal Nitrogen		Zinc
	Phosphorus		
	Phosphate		
	Nitrite		
	Nitrate		
	Sulfide		

**Table 2:** Comparison of standard deviation for original data and after log transformation.

Parameters	Standard Deviation for Original Data	Standard Deviation for Data Transformation
pH	1.088	0.088
Temperature	1.011	0.015
Total solid	2306.153	0.508
TDS	2121.007	0.882
Turbidity	60.605	0.695
Electrical Conductivity	4887.621	0.565
Salinity	2.380	0.511
Dissolved oxygen	0.952	0.155
Phosphorous	0.470	0.603
Ammonia	5.594	0.434
Ammoniacal nitrogen	10.008	0.394
Phosphate	4.139	0.728
Nitrate	9.871	0.710
Nitrite	0.108	0.077
Sulfide	0.093	0.450
BOD	0.224	0.083
COD	21.834	0.176
Magnesium	103.282	0.623
Calcium	31.397	0.494
Potassium	38.970	0.361
Sodium	601.028	0.593
Chloride	1286.808	0.757
Bicarbonate	388.351	0.515
Sulfate	181.464	1.158
Aluminium	3.698	0.725
Arsenic	0.142	0.074
Barium	0.505	0.167
Cadmium	0.105	0.020
Copper	0.146	0.054
Iron	18.754	0.654
Manganese	2.465	0.594
Strontium	0.418	0.044
Silica	13.546	0.281
Zinc	0.188	0.065

**Table 3:** Not normal data tabulation before normality testing.

One-Sample Kolmogorov-Smirnov Test		Magnesium
N		109
Normal parameters	Mean	58.89
Most Extreme Differences	Std. Deviation	103.28
	Absolute	0.314
	Positive	0.314
	Negative	−0.285
Kolmogorov-Smirnov Z		3.284
*p*-value		0.00

**Table 4:** Not normal data tabulation before normality testing.

One-Sample Kolmogorov-Smirnov Test		Magnesium
N		109
Normal parameters	Mean	1.32
Most Extreme Differences	Std. Deviation	0.62
	Absolute	0.07
	Positive	0.07
	Negative	−0.067
Kolmogorov-Smirnov Z		0.727
*p*-value		0.666

**Table 5:** The values of the total variance and rotated components matrix for preliminary analysis.

Component	Eigenvalues	% of Variance	Cumulative %	Components Matrix after Varimax Rotations		
				Parameters	1st Components	2nd Components
1	8.841	26.004	26.004	LogpH	0.475	−0.730
2	6.206	18.254	44.258	LogTemperature	−0.150	−0.606
3	3.639	10.704	54.962	LogTS	0.714	0.658
4	2.360	6.941	61.903	LogTDS	0.223	0.890
5	1.939	5.702	67.604	LogTurbidity	0.109	0.687
6	1.367	4.021	71.625	LogEC	0.927	0.099
7	1.189	3.496	75.121	LogSalinity	0.816	0.194
8	0.940	2.764	77.885	LogDO	−0.011	0.694
9	0.855	2.514	80.399	LogBOD	0.015	0.658
10	0.818	2.405	82.804	LogCOD	0.684	0.049
11	0.643	1.890	84.694	LogBicarbonate	0.414	0.565
12	0.630	1.854	86.548	LogChloride	0.919	−0.033
13	0.592	1.740	88.288	LogSilica	0.079	0.729
14	0.501	1.472	89.760	LogPhosporous	0.275	0.648
15	0.480	1.411	91.170	LogAmmonia	0.461	−0.276
16	0.390	1.146	92.316	LogAN	0.357	0.827
17	0.365	1.075	93.391	LogSulfate	−0.177	0.602
18	0.322	0.947	94.338	LogPhosphate	0.163	0.826
19	0.273	0.802	95.140	LogNitrate	0.120	0.825
20	0.258	0.759	95.899	LogNitrite	0.063	0.840
21	0.234	0.689	96.588	LogSulfide	0.093	0.800
22	0.188	0.552	97.140	LogAl	0.086	0.574
23	0.174	0.511	97.651	LogAs	0.090	0.697
24	0.143	0.420	98.071	LogBarium	0.088	0.713
25	0.139	0.408	98.479	LogCalcium	0.631	0.236
26	0.110	0.323	98.802	LogCadmium	−0.079	0.804
27	0.084	0.249	99.050	LogIron	−0.174	0.520
28	0.073	0.213	99.264	LogPotassium	0.835	0.063
29	0.070	0.205	99.468	LogMagnesium	0.755	0.032
30	0.062	0.182	99.651	LogManganese	0.109	0.733
31	0.048	0.141	99.792	LogSrontium	0.660	−0.108
32	0.041	0.119	99.911	LogZinc	−0.053	0.791
33	0.024	0.071	99.982	LogCopper	0.107	0.812
34	0.006	0.018	100.000	LogSodium	0.862	−0.114

Extraction Method: Principal Component Analysis. Rotation Method: Varimax with Kaiser Normalization.

**Table 6:** The values of total variance and rotated components matrix for final analysis.

Component	Eigenvalues	% of Variance	Cumulative %	Components Matrix after Varimax Rotations		
				Parameters	1st Components	2nd Components
1	6.934	43.336	43.336	LogEC	0.919	0.058
2	3.466	21.663	64.999	LogChloride	0.901	0.842
3	1.367	8.545	73.544	LogPotassium	0.868	0.751
4	1.029	6.434	79.978	LogSodium	0.861	0.877
5	0.681	4.258	84.236	LogCOD	0.731	0.899
6	0.539	3.371	87.606	LogMagnesium	0.721	0.264
7	0.488	3.049	90.655	LogCalcium	0.632	0.509
8	0.393	2.459	93.114	LogSalinity	0.559	0.771
9	0.270	1.689	94.802	LogTS	0.624	0.742
10	0.239	1.494	96.296	LogTDS	−0.083	0.950
11	0.200	1.248	97.544	LogAmmonia	0.406	0.542
12	0.164	1.026	98.570	LogAl	0.131	0.793
13	0.102	0.635	99.204	LogCadmium	0.424	0.868
14	0.067	0.417	99.621	LogCopper	0.344	0.841
15	0.052	0.327	99.948	LogZinc	0.060	0.889
16	0.008	0.052	100.000	LogBOD	0.378	0.803

Extraction Method: Principal Component Analysis. Rotation Method: Varimax with Kaiser Normalization.

**Table 7:** Dominant parameters from final PCA analysis.

Subgroups of Parameters	Dominant Parameters
Physicochemical Characteristic	Electrical conductivity
Aggregate Indicator	Chemical oxygen demand
Major Ions	Magnesium, calcium, potassium, sodium, and chloride

**Table 8:** The minimum and the maximum limits value of groundwater from different references.

Parameters	Min Limit of Groundwater in Forest (mg/L)	Max Limit (mg/L)	References	Mean of Parameters Concentration (mg/L)
Magnesium	1	150	MOH (2004)	79.50
Calcium	1	200	UNEP (2013)	18.02
Potassium	2	100	UNEP (2013)	28.76
Sodium	5	1000	UNEP (2013)	403.84
Chloride	5	1000	BIS (2012)	731.60
Electrical Conductivity (µs/cm)	20	10,000	APHA (2000)	2889.40 (µs/cm)
COD	10	200	UNEP (2013)	57.00

**Table 9:** Subindex value of parameters using proportional analysis.

Parameters	Concentration Range	Subindex
**Magnesium**	x ≤ 1 150−1=149 $\frac{150-27.50}{149}\times 100=47$ x ≥ 150	47
**Calcium**	x ≤ 1 200−1=199 $\frac{200-18.02}{199}\times 100=92$ x ≥ 200	92
**Potassium**	x ≤ 2 100−2=98 $\frac{100-28.80}{98}\times 100=73$ x ≥ 100	73
**Sodium**	x ≤ 5 1000−5=995 $\frac{1000-403.84}{995}\times 100=60$ x ≥ 1000	60
**Chloride**	x ≤ 5 1000−5=995 $\frac{1000-731.60}{995}\times 100=27$ x ≥ 1000	27
**Electrical Conductivity**	x ≤ 20 10,000−20=9980 $\frac{10,000-2889.40}{9980}\times 100=71$ x ≥ 10,000	71
**Chemical Oxygen Demand**	x ≤ 10 200−10=190 $\frac{200-57.00}{190}\times 100=75$ x ≥ 200	75

**Table 10:** Interquartile range (IQR) calculation for each index indicator.

Parameters		Mg	Ca	K	Na	Chloride	EC	COD
**N**	Valid Missing	109	109	109	109	109	109	109
		0	0	0	0	0	0	0
**Percentiles**	(Q1) 25	6.8	5.55	10.27	40.0	51.1	382.0	44.45
	50	23	9.86	14.0	105.30	169.43	852.5	46.54
	(Q3) 75	48.26	17.5	22.5	195.89	355	1936.0	51.43
**IQR values**	20	41.46	11.95	12.23	155.89	303.9	1554	6.98

**Table 11:** Limit of the range of index scores for all subindexes.

Classes	Range of Index Scores							
	Mg	Ca	K	Na	Chloride	EC	COD	Average
**I**	100	100	100	100	100	100	100	100
**II**	72	70	67	69	70	69	70	69
**III**	45	40	35	38	40	38	37	39
**IV**	17	10	2	6	9	7	6	9

**Table 12:** The distinct classes according to the range of index scores.

Classes	Range of Index Scores
I	70–100
II	40–69
III	10–39
IV	0–9

**Table 13:** Groundwater classification for the index value.

Classes	Range of Index Scores	Groundwater Classification
**I**	70–100	Excellent
**II**	40–69	Good
**III**	10–39	Fair
**IV**	0–9	Poor

**Table 14:** Groundwater description for each groundwater classification.

Classes	Range of Index Scores	Groundwater Classification
**Excellent**	70–100	Very clean, suitable for domestic utilization with the standard water treatment process before use.
**Good**	40–69	Clean, suitable for domestic utilization with the standard treatment process before use.
**Fair**	10–39	Fairly clean, suitable for domestic utilization however requires an intensive water treatment process before use.
**Poor**	0–9	Groundwater sources are unsuitable for domestic utilization.

**Table 15:** Subindex calculation by concentration increment.

Parameters	Concentration Range
**Magnesium**	x ≤ 1 150−1=149 $\frac{150-200}{149}\times 100=33.56$ x ≥ 150

**Table 16:** Subindex calculation by concentration decrement.

Parameters	Concentration Range
**Magnesium**	x ≤ 1 150−1=149 $\frac{150-0.24}{149}\times 100=100$ x ≥ 150

## References

[1] Connor R. (2015). The United Nations World Water Development Report 2015: Water for a Sustainable World.

[2] Sefie A., Aris A.Z., Ramli M.F., Narany T.S., Shamsuddin M.K.N., Saadudin S.B., Zali M.A. (2018). Hydrogeochemistry and groundwater quality assessment of the multilayered aquifer in Lower Kelantan Basin, Kelantan, Malaysia. Environ. Earth Sci..

[3] Asadi E., Isazadeh M., Samadianfard S., Ramli M.F., Mosavi A., Nabipour N., Shamshirband S., Hajnal E., Chau K.W. (2020). Groundwater quality assessment for sustainable drinking and irrigation. Sustainability.

[4] FAO (2018). The State of Food and Agriculture 2018. Migration, Agriculture and Rural Development.

[5] United Nations (2014). Human Development Report 2014: Sustaining Human Progress-Reducing Vulnerabilities and Building Resilience.

[6] Lee K.E., Mokhtar M., Mohd Hanafiah M., Abdul Halim A., Badusah J. (2016). Rainwater harvesting as an alternative water resource in Malaysia: Potential, policies and development. J. Clean. Prod..

[7] Rivett M.O., Symon S., Jacobs L., Banda L.C., Wanangwa G.J., Robertson D.J.C., Hassan I., Miller A.V.M., Chavula G.M.S., Songola C.E. (2020). Paleo-geohydrology of lake chilwa, malawi is the source of localised groundwater salinity and rural water supply challenges. Appl. Sci..

[8] Razak Y.A., Mohammed Hatta A. (2009). Groundwater Management in Malaysia-Status and Challenges. Proc. Groundw. Colloqium 2009.

[9] Sun W., Xia C., Xu M., Guo J., Sun G. (2016). Application of modified water quality indices as indicators to assess the spatial and temporal trends of water quality in the Dongjiang River. Ecol. Indic..

[10] Kachroud M., Trolard F., Kefi M., Jebari S., Bourrié G. (2019). Water quality indices: Challenges and application limits in the literature. Water.

[11] Horton R. (1965). An Index Number System for Rating Water Quality. J. Water Pollut. Control Fed..

[12] Brown R.M., McClelland N.I., Deininger R.A., Tozer R.G. (1970). A water quality index: Do we dare?. Water Sew. Work..

[13] Steinhart C.E., Schierow L.-J., Sonzogni W.C. (1982). An Environmental Quality Index for the Great Lakes. J. Am. Water Resour. Assoc..

[14] Kura N.U., Ramli M.F., Sulaiman W.N.A., Ibrahim S., Aris A.Z. (2015). An overview of groundwater chemistry studies in Malaysia. Environ. Sci. Pollut. Res..

[15] Ewaid S.H., Abed S.A., Al-ansari N., Salih R. (2020). Development and Evaluation of a Water Quality Index for the Iraqi Rivers. Hydrology.

[16] Sahoo M.M., Patra K.C., Khatua K.K. (2015). Inference of Water Quality Index using ANFIA and PCA. Aquat. Procedia.

[17] Mahapatra S.S., Sahu M., Patel R.K., Panda B.N. (2012). Prediction of Water Quality Using Principal Component Analysis. Water Qual. Expo. Health.

[18] Abbasi T., Abbasi S.A. (2012). Water Quality Indices.

[19] Isa N.M., Aris A.Z., Lim W.Y., Sulaiman W.N.A.W., Praveena S.M. (2014). Evaluation of heavy metal contamination in groundwater samples from Kapas Island, Terengganu, Malaysia. Arab. J. Geosci..

[20] Aris A.Z., Harun A.M., Woong K.K., Mangala P.S. Compositional Change of Groundwater Chemistry in the Shallow Aquifer of Small Tropical Island Due to Seawater Intrusion. Proceedings of the 20th Salt Water Intrusion Meeting.

[21] Brodie R., Sundaram B., Tottenham R., Hostetler S., Ransley T. (2007). An Overview of Tools for Assessing Groundwater-Surface Water Connectivity. Bur. Rural Sci. Canberra.

[22] Reyes-Toscano C.A., Alfaro-Cuevas-Villanueva R., Cortés-Martínez R., Morton-Bermea O., Hernández-Álvarez E., Buenrostro-Delgado O., Ávila-Olivera J.A. (2020). Hydrogeochemical characteristics and assessment of drinkingwater quality in the Urban Area of Zamora, Mexico. Water.

[23] Bartolino J.R., Cole J.C., US Geological Survey (2002). Ground-Water Resources of the Middle Rio Grande Basin, New Mexico.

[24] Kura N.U., Ramli M.F., Ibrahim S., Sulaiman W.N.A., Aris A.Z., Tanko A.I., Zaudi M.A. (2014). Assessment of groundwater vulnerability to anthropogenic pollution and seawater intrusion in a small tropical island using index-based methods. Environ. Sci. Pollut. Res..

[25] Kura N.U., Ramli M.F., Sulaiman W.N.A., Ibrahim S., Aris A.Z., Mustapha A. (2013). Evaluation of factors influencing the groundwater chemistry in a small tropical Island of Malaysia. Int. J. Environ. Res. Public Health.

[26] Rahim B.E., Yusoff I., Samsudin R., Yaacob W.Z.W., Rafek G.M. (2010). Deterioration of groundwater quality in the vicinity of an active open-tipping site in West Malaysia. Hydrogeol. J..

[27] Aris A.Z., Abdullah M.H., Ahmed A., Woong K.K. (2007). Controlling factors of groundwater hydrochemistry in a small island’s aquifer. Int. J. Environ. Sci. Technol..

[28] Sefie A., Aris A.Z., Shamsuddin M.K.N., Tawnie I., Suratman S., Idris A.N., Saadudin S.B., Wan Ahmad W.K. (2015). Hydrogeochemistry of Groundwater from Different Aquifer in Lower Kelantan Basin, Kelantan, Malaysia. Procedia Environ. Sci..

[29] American Public Health Association, American Water Works Association, Water Environment Federation (2012). Standard Methods for Examination of Water and Wastewater.

[30] Appelo C.A.J., Postma D. (2005). Geochemistry, Groundwater and Pollution.

[31] Scheili A., Rodriguez M.J., Sadiq R. (2015). Development, application, and sensitivity analysis of a water quality index for drinking water management in small systems. Environ. Monit. Assess..

[32] Tomaszkiewicz M., Abou Najm M., El-Fadel M. (2014). Development of a groundwater quality index for seawater intrusion in coastal aquifers. Environ. Model. Softw..

[33] Saeedi M., Abessi O., Sharifi F., Meraji H. (2010). Development of groundwater quality index. Environ. Monit. Assess..

[34] Stigter T.Y., Ribeiro L., Carvalho Dill A.M.M. (2006). Application of a groundwater quality index as an assessment and communication tool in agro-environmental policies—Two Portuguese case studies. J. Hydrol..

[35] Atta M., Yaacob W.Z.W., Jaafar O.B., Sakawi Z. (2014). Bin Ammoniacal nitrogen contaminated groundwater at taman beringin ex–landfill site: Implication to health and the environment. Adv. Environ. Biol..

[36] Hair J.F., Black W.C., Babin B.J., Anderson R.E. (2010). Multivariate Data Analysis.

[37] Rencher A.C. (2002). Methods of Multivariate Analysis.

[38] WHO (2012). Guidelines for Drinking Water Quality.

[39] U.S.E.P.A (2014). Drinking Water Parameters Microbiological, Chemical and Indicator Parameters in the 2014 Drinking Water.

[40] Bureau of Indian Standards BIS (2012). Indian Standard for Drinking Water Specifications. Second Revision.

[41] UNEP (2013). Groundwater and its Susceptibility to Degradation: A Global Assessment of the Problem and Options for Management.

[42] Zhang B., Zhao D., Zhou P., Qu S., Liao F., Wang G. (2020). Hydrochemical characteristics of groundwater and dominantwater-rock interactions in the Delingha area, Qaidam Basin, Northwest China. Water.

[43] Shin K., Koh D.C., Jung H., Lee J. (2020). The hydrogeochemical characteristics of groundwater subjected to seawater intrusion in the Archipelago, Korea. Water.

[44] Jeong J., Jeen S.-W., Hwang H.-T., Lee K.-K. (2020). Changes in Geochemical Composition of Groundwater Due to CO2 Leakage in Various Geological Media. Water.

[45] Nolan B., Stoner J. (2000). Nutrients in Groundwaters of the Conterminous United States, 1992–1995. Environ. Sci. Technol..

[46] Klimaszyk P., Rzymski P., Piotrowicz R., Joniak T. (2014). Contribution of surface runoff from forested areas to the chemistry of a through-flow lake. Environ. Earth Sci..

[47] Allen A., Chapman D. (2001). Impacts of afforestation on groundwater resources and quality. Hydrogeol. J..

[48] Chen L., Li C., Xu B., Xing B., Yi G., Huang G., Zhang C., Liu J. (2019). Microbial degradation of organic pollutants in groundwater related to underground coal gasification. Energy Sci. Eng..

[49] Das N., Chandran P. (2011). Microbial Degradation of Petroleum Hydrocarbon Contaminants: An Overview. Biotechnol. Res. Int..

[50] DOA (2005). Malaysian Farm Certification Scheme for Good Agricultural Practice.

[51] Han J., Kamber M., Pei J. (2012). Data Mining Concepts and Techniques.

[52] MWA (2014). The Malaysian Water Association Quarterly.

[53] Tiwari A.K., De Maio M., Singh P.K., Singh A.K. (2016). Hydrogeochemical characterization and groundwater quality assessment in a coal mining area, India. Arab. J. Geosci..

[54] Khaki M., Yusoff I., Islami N. (2016). Electrical resistivity imaging and hydrochemical analysis for groundwater investigation in Kuala Langat, Malaysia. Hydrol. Sci. J..

[55] Harun H.H., Mohamad Roslan M.K., Nurhidayu S., Ash’aari Z.H., Kusin F.M. (2021). Analyzing the major ions and trace elements of groundwater wells in Kuala Langat, Selangor. Malays. J. Fundam. Appl. Sci..

[56] Harun H.H., Mohamad Roslan M.K., Nurhidayu S., Ash’aari Z.H., Kusin F.M. (2021). Physicochemical analysis of groundwater and suitability for domestic utilization in Kuala Langat, Selangor. Int. Conf. Civil Environ. Eng..

